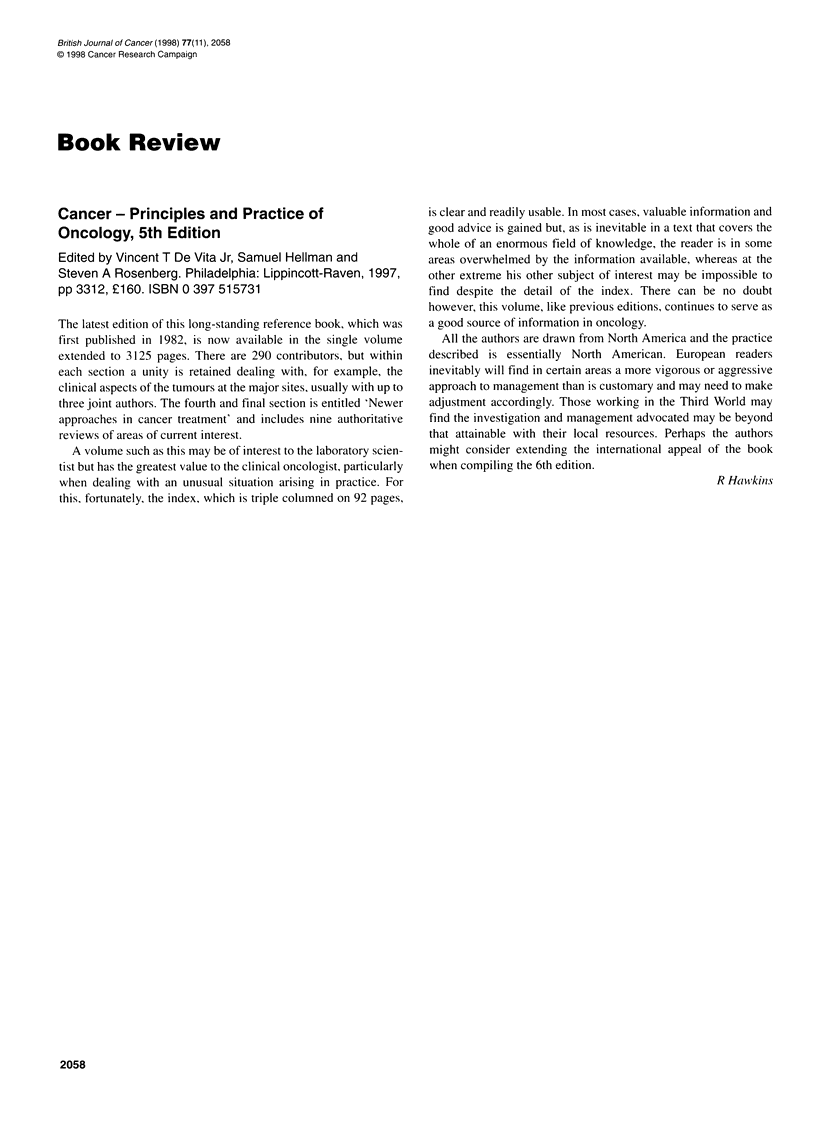# Cancer - Principles and Practice of Oncology, 5th Edition

**Published:** 1998-06

**Authors:** R Hawkins


*British Journal of Cancer* (1998) **77**(11), 2058
© 1998 Cancer Research Campaign

### Book Review

### Cancer – Principles and Practice of Oncology, 5th Edition

Edited by Vincent T De Vita Jr, Samuel Hellman and Steven A Rosenberg. Philadelphia: Lippincott-Raven, 1997, pp 3312, £160. ISBN 0 397 515731

The latest edition of this long-standing reference book, which was first published in 1982, is now available in the single volume extended to 3125 pages. There are 290 contributors, but within each section a unity is retained dealing with, for example, the clinical aspects of the tumours at the major sites, usually with up to three joint authors. The fourth and final section is entitled 'Newer approaches in cancer treatment' and includes nine authoritative reviews of areas of current interest.

A volume such as this may be of interest to the laboratory scientist but has the greatest value to the clinical oncologist, particularly when dealing with an unusual situation arising in practice. For this, fortunately, the index, which is triple columned on 92 pages, is clear and readily usable. In most cases, valuable information and good advice is gained but, as is inevitable in a text that covers the whole of an enormous field of knowledge, the reader is in some areas overwhelmed by the information available, whereas at the other extreme his other subject of interest may be impossible to find despite the detail of the index. There can be no doubt however, this volume, like previous editions, continues to serve as a good source of information in oncology.

All the authors are drawn from North America and the practice described is essentially North American. European readers inevitably will find in certain areas a more vigorous or aggressive approach to management than is customary and may need to make adjustment accordingly. Those working in the Third World may find the investigation and management advocated may be beyond that attainable with their local resources. Perhaps the authors might consider extending the international appeal of the book when compiling the 6th edition.

*R Hawkins*